# Drug Therapy For Preventing Ventricular Arrhythmia In Brugada syndrome: Do We Have The Answers Yet?

**DOI:** 10.1016/s0972-6292(16)30667-2

**Published:** 2013-09-01

**Authors:** Shomu Bohora

**Affiliations:** U N Mehta Institute of Cardiology and Research Centre. Ahmedabad.

**Keywords:** Drug Therapy, Brugada syndrome, Ventricular Arrhythmia

After the first description of Brugada syndrome [1] and its genetic mutation in SCN5A gene [2], the understanding of this, often fatal, genetic abnormality has greatly increased. Electrophysiologically, ventricular myocardium is composed of three distinct cell types, namely epicardial, endocardial, and M cells [3]. Action potentials recorded from epicardial and M cells display a spike-and-dome morphology, because of prominent transient outward current-mediated phase 1, which is not prominent in the endocardial cells. In Brugada syndrome, different genetic mutations in the SCN5A gene, which encodes for the α subunit of the cardiac sodium channel, causes a loss of function in the I_Na_ channel, resulting in a loss of the action potential dome in epicardial myocytes. Accentuated I_to_-mediated action potential notch and the loss of action potential dome in the epicardium, but not endocardium of the right ventricular outflow tract, gives rise to a transmural voltage gradient. This causes ST segment elevation in leads V1 - V3 and induction of ventricular fibrillation (VF) due to phase 2 reentry [4-8].

Agents that reduce outward currents (e.g. I_to_, I_K-ATP_, I_Ks_, I_Kr_) or boost inward currents (e.g. I_Ca-L_) at the end of phase 1 of AP can attenuate ST segment elevation and suppress episodes of ventricular arrhythmia. Such agents are candidates for a pharmacological approach to therapy of the Brugada syndrome [9]. In right ventricular wedge preparations [6,8], exposure to pinacidil (potassium channel opener); a combination of pilsicainide (class IC sodium channel blocker) and terfenadine (I_Ca-L_ blocker); and acetylcholine, a parasympathetic agonist (blocking effect of I_Ca-L_), produces a transmural voltage gradient between epicardial and endocardial cells and ST segment elevation. Isoproterenol (β-adrenergic agonist) strongly augments I_Ca-L_ and restores the epicardial dome thus decreasing the ST segment elevation. I_to_ blockers like 4-aminopyridine (relatively selective), tedisamil, and quinidine (less selective), also restore the epicardial dome and decrease phase 1 action potential notch, thus decreasing ST segment elevation. In experimental settings the episodes of ventricular arrhythmia induced, were shown to be successfully suppressed by I_to_ blockade [6,8].

Based on the cellular mechanism responsible for Brugada phenotype, a cardio-selective I_to_ blocker would be an ideal pharmaceutical approach. Efficacy of drugs in patients with Brugada syndrome to prevent ventricular arrhythmia [9-20], especially quinidine, as reported by Stelios P et al in this journal [21], has been well documented in literature. Quinidine decreases the transmural dispersion by blocking repolarizing currents, specifically I_to_ channel, and hence has shown to be clinically effective in episodes of ventricular storm [9,10] along with isoproterenol [9]. Denopamine (oral adrenergic stimulant) and atropine (anti-cholinergic agent) increases I_Ca-L_ and are also shown to be effective [11]. Cilostazol, a phosphodiesterase III inhibitor, also has shown to reduce ST segment elevation, probably due to its effect to increase I_Ca-L_ and heart rate [12].

Hermida et al [16] also showed that hydroquinidine therapy prevented ventricular tachycardia (VT)/VF inducibility in 76% of asymptomatic patients with Brugada syndrome on electrophysiology study, as well as VT/VF recurrence in all patients with multiple ICD shocks and led the authors to suggest that preventive treatment by hydroquinidine may be an alternative strategy to implantable cardiac defibrillator (ICD) placement in asymptomatic patients with Brugada syndrome and inducible arrhythmia. In another study by Belhassen et al [17], class IA drugs, especially quinidine, effectively prevented induction of VT/VF in 96% patients. In their study of 23 patients treated with these medications, no patient died or had a sustained ventricular arrhythmia during a mean follow-up period of 9.1 +/- 5.6 years (7 to 20 years in 15 patients) which suggested that EP-guided therapy, with Class IA agents is a reasonable, safe, and effective approach for the long-term management of patients with idiopathic VF.

With increasing utilization of non-drug approaches in Brugada syndrome, such as ICD's and ablation therapy [20], use of antiarrhythmic drugs has not been uniformly recommended and there exists no large randomized prospective studies of effectiveness of drugs, especially quinidine, in patients having Brugada syndrome with/without ICD's. However, quinidine is the only oral antiarrhythmic agent, which consistently seems to be effective in treatment of ventricular arrhythmia related to Brugada syndrome [18-20]. However, quinidine as an antiarrhythmic drug, is not very popular as it requires frequent dosing, is associated with frequent side effects and is not considered as effective as other class III antiarrhythmic agents. Hence the availability of quinidine decreased, as manufacturers found the drug production and marketing non-viable and stopped manufacturing them [22]. Orphan-drug status is intended for drugs that treat fewer patients, and the company, on its development is not expected to recover the cost of developing and marketing the drug, but needs to invest, due to social and political responsibilities. Quinidine doesn't even fit into this category, as it is already a developed drug.

Although quinidine is one of the oldest drugs, there has been renewed interest in quinidine, for its effectiveness in treating ventricular arrhythmia related to Brugada syndrome, and with fewer options available till the time a specific I_to_ blocker is available for clinical use, quinidine will continue to have a role in modern cardiology. Hence, drug manufacturing companies should take social responsibility, and make this "endangered species" drug, easily available [21,22].
